# Professor Christison’s Lecture on the Present State of Medical Evidence

**Published:** 1852-06

**Authors:** 


*Prof. Christison's Lecture on the present state of Medical Evidence.*—The first objection to be stated against it (the practice of not allowing medical witnesses to be present in court to hear the evidence of the general witnesses) is, that it tends to suppress and conceal medical facts. It is impossible for judge or counsel to know always what facts in the general evidence are medical, and what not. No one will doubt the aptitude with which the lawyer acquires for the occasion a knowledge of the particular art or science concerned in a law case. But it is not to be supposed that any aptitude or any practice of the kind will enable him to single out from the testimony of the general witnesses the whole facts which bear upon medical opinion. It has been already mentioned, that one of the shrewdest lawyers who ever occupied the Scottish bench once made this admission in court. I may now mention a remarkable illustration of its justice. In 1831, on the trial relative to the disputed insurances on the life of the late Earl of Mar, which it was maintained had been viti ated by concealment of the vice of opium-eating, the presiding judge, the late Lord Chief Commissioner Adam, told the jury in his charge, as a fact in the case, that although there was evidence of the Earl having purchased laudanum at the rate of nearly three ounces a-day for some years, there was no evidence of his having actually taken it to that excess. There was indeed no direct evidence of his having done so. But the judge, not being a medical man, had not perceived the medical bearing of a most pregnant little fact which was sworn to by the Earl's housekeeper; who said she gave him every morning before he got up so large a dose as a tablespoonful of laudanum at once. As he was also proved to have taken habitually several other doses during the day, the whole quantity was approximately accounted for. But—which is still more pointed—the enormous dose, and the time of day when he took it, were to every medical man irrefragable proof that Lord Mar must have long been a practised and slavish opium-eater.—[Med. and Surg. Journal, xxxvii., 130.]

Another objection is, that facts are liable to be misstated and misrepresented, owing to errors of language. One of the chief reasons for the preference we give to the direct testimony of our own senses over the testimony of others, is the risk of error from the necessity of the intervening use of language. The medical witness is subjected by the present rule of Scotch courts to a two-fold risk of this kind, inasmuch as he receives the facts, not directly from him who witnessed and describes them, but through the intervention of a third party. And the risk of error is all the greater, that many words have a double meaning, one general, and the other scientific or professional. Some years ago, on an important trial in the High Court of Justiciary for assault, the public prosecutor attempted to prove, that the person assailed had been wounded to the effusion of his blood; which is held in law to be an aggravation of guilt in such cases. When the principal medical witness was examined as to the injuries inflicted, he was asked whether any blood had been effused; and he replied that a good deal must have been effused. But he meant that there was effusion of blood under the skin, constituting the contusion he had described; while the counsel and court at first received his answer as implying that there had been considerable loss of blood from a wound. The latter view was on the point of passing to the jury as a fact, when one of the judges detected the equivoque, and set the matter to rights. But the incident is not the less illustrative of the risk of serious error, in circumstances in which scarcaly any medical man could have gone wrong, had he been duly pre-informed as to the bearings of his evidence.

A third objection is, that the medical witness has no opportunity of judging of the truth of the facts, on which he is asked to found his opinion. The same professional skill, which enables him to collect the facts better than another, makes him also a better judge of their authenticity. I am aware of the theory in law, which holds that the jury alone are to judge of the truth of the facts. I apprehend, however, this doctrine may be meant to apply only to those facts whose truth depends on the veracity of the witness. But at any rate, both in regard to such facts, and all others indiscriminately, the rule is violated by the present practice of Scotch courts in examining medical witnesses. The facts do not pass to them with the stamp of authentication from the jury. For the questions put to the witnesses are framed from the facts, and put generally by the counsel, and occasionally by the judge, but very seldom by the jury. In truth, the rule must be inevitably violated, whenever evidence consists of opinion from facts deposed to by others; because no man in such circumstances can either form an opinion as a witness, or shape questions to elicit an opinion from a witness, without exercising his judgment as to the authenticity of the facts. I do not here refer so much to facts whose authenticity depends on the veracity of the witness who deposes to them. His evidence as to facts, whence medical opinions are to be deduced, may be morally true, and nevertheless scientifically false. And it is mainly their authenticity in the latter respect, which is referred to at present, as a matter which must be determined before an opinion can be safely formed from them. According to the existing practice of Scotch courts, this is done by the counsel and the judge. Is it not more rational that it should be done by the witness himself, who alone is qualified by professional education to exercise such judgment?

No one can hesitate how to answer that question, who is acquainted with the nature of medical facts—the acuteness required for observing them—the experience for appreciating them—the judgment for distinguishing fact from opinion. I would beg in particular to remark, that few are aware, and no unprofessional person can be adequately so, of the great risk that opinion shall be mistaken for fact.

Facts being mostly cognizable by the cxternal senses, they are received in evidence much more unreservedly than opinions; which, besides the exercise of observation, involve that of many other faculties of the mind, all much more liable to error. Hence opinions, if taken for facts, will be apt to be admitted as such with undue facility, because not duly tested by the judgment. Spurious facts of this sort abound in pure medicine. How much more then in medical jurisprudence, if supplied by unpıofessional persons! Suppose that in a trial on account of a disputed assurance, an ordinary witness deposes that the party insured had at a certain date palsy in the limbs. To a non-medical eye nothing could be liker a simple fact. But it is no such thing. The affection may have been lameness, left by an old rheumatism. It may depend on stiffness of one or more joints from local disease or injuries; it may be part only of a general debility, affecting, as it often does, the limbs more than the rest of the body; it may be a mere awkwardness of gait. In calling the affection palsy, then, the witness does not state a fact, he forms a diagnosis, he delivers an opinion, and an opinion very likely to be wrong; for it is sometimes no easy matter, even for a medical man, to make up his mind as to the existence of paraplegia in its early stage. How often upon trials does it happen, that in describing the appearances in suspected poisoning, the witness says he saw inflammation of the stomach! and this is received as a statement of fact. But is it really such? Several characters are necessary to constitute inflammation: some of these may be imitated by the state of the stomach at certain stages of digestion; others by pseudo-morbid appearaces arising spontaneously a short time before or after death; others by the action of extraneous agents subsequently. The witness, therefore, does not describe a simple fact: he forms a pathological opinion. And were any further proof necessary to establish this, I have but to remind you how often he is discovered to be wrong. Some years ago a man in London was tried for murdering his mistress, and throwing the body into the Thames. His defence was that they determined to drown themselves together; but that his resolution gave way in the water; and that, while he with difficulty saved himself, he failed in his efforts to save also his companion. But three surgeons, in this matter no wiser than three ordinary observers, swore at the coroner's inquest, that they found contusions on the head and face of the girl's dead body. This passed for a fact, and to most unprofessional persons would seem a very simple fact. The man was accoadingly tried for his life. But on the trial a fourth surgeon swore, that he afterwards dissected the alleged bruises, found no traces of congested or extravasated blood, and that in his opinion the appearances were no more than the livid marks which are apt to arise in many bodies after death. A somewhat similar case occurred in this city some years before, in which the prisoner justly escaped through the evidence of the late Mr. John Bell and Mr. Fyfe.

If I may trust my own experience of proceedings in criminal and civil trials, the confounding of medical opinion with medical fact is a matter of very frequent occurrence, and one serious cause of the imperfections of medical evidence. Nor is it rashness of criticism to express a fear, that errors originating in this cause must be very apt to happen under the present system, which requires medical witnesses to form their opinion on the facts, without a previous opportunity of sifting them.

A fourth objection is, that the medical witness can have no correct idea of the bearings of his evidence on the case. This is too evident to need illustration. The proposition indeed may with reason be stated more strongly. For he will be very apt to take up an incorrect idea of the bearing of his evidence; inasmuch as questions framed upon general propositions, or supposed conditions, can not fail to give him some insight, and yet a very imperfect and probably incorrect one, into the ordinary facts and nature of the case.

